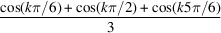# Form factor of helical structures and twisted fibres

**DOI:** 10.1107/S1600576723008671

**Published:** 2023-11-07

**Authors:** Johan R. C. van der Maarel

**Affiliations:** a Department of Physics, National University of Singapore, 117542 Singapore; Lund University, Sweden; Keele University, United Kingdom

**Keywords:** helical filaments, helical fibres, small-angle scattering, form factors, twisted hexagonal fibres

## Abstract

A general formalism is presented for the isotropically averaged single-chain scattering function (form factor) of single, double, triple and higher-order helices, as well as twisted fibres consisting of concentric layers of strands. Form factors for double and triple helices with differently sized grooves have also been derived. The formulas include the longitudinal and transverse interference over the pitch and radius of the helices, respectively. The results may be useful for the analysis of small-angle scattering data of (bio)macromolecules or molecular assemblies exhibiting a helical arrangement.

## Abstract 2

A general formalism is presented for the single-chain scattering function of helices and twisted fibres with easy adaptation of number of strands and cross-sectional symmetry.

## 1. Introduction

Helical diffraction theory was first developed for atoms on a regular helix and used to solve the molecular structure of nucleic acids (Cochran *et al.*, 1952 ▸; Wilkins *et al.*, 1953 ▸). Expressions for the isotropically averaged single-chain scattering function (form factor) have been reported for helical filaments, pairs of coaxial helical filaments, pairs of coaxial strands of different cross section and helical ribbons (Schmidt, 1970 ▸; Pringle & Schmidt, 1971 ▸; Puigjaner & Subirana, 1974 ▸; Muroga, 2001 ▸; Hamley, 2008 ▸). The form factor of a superhelix consisting of two diametrically opposed strands has been derived and used for the analysis of DNA supercoiling (Zakharova *et al.*, 2002 ▸; Zhu *et al.*, 2010 ▸). In the present contribution, the form factors of various helical structures are derived and cast in a general formalism for easy adaptation of the number of coaxial strands and cross-sectional symmetry. In particular, formulas are reported for double and triple helices with grooves of different widths, as well as twisted fibres consisting of concentric layers of strands. The results refer to continuous helices and do not include spacing of individual atoms or groups of atoms at regular intervals. The derived formulas may hence be useful for the analysis of low-resolution scattering data of randomly or partially oriented (bio)macromolecules or molecular assemblies exhibiting a helical structural arrangement.

This article is organized as follows. First, the form factor of a single helix is derived. The single-helix results provide the basis for the derivation of the form factor of multiple-order helices. Second, the form factor of a double helix is presented, both for the symmetric case with opposing strands and for the non-symmetric case with a major and minor groove. Third, the same analysis is applied to a triple helix, including the presence of differently sized grooves. Fourth, the form factor pertaining to a higher-order helix with any number of equally spaced strands but at a fixed radial distance from the central axis is derived. Fifth, the form factor of hexagonal twisted fibres consisting of concentric layers of parallel strands is presented.

## 2. Single helix

**Figure 1:**
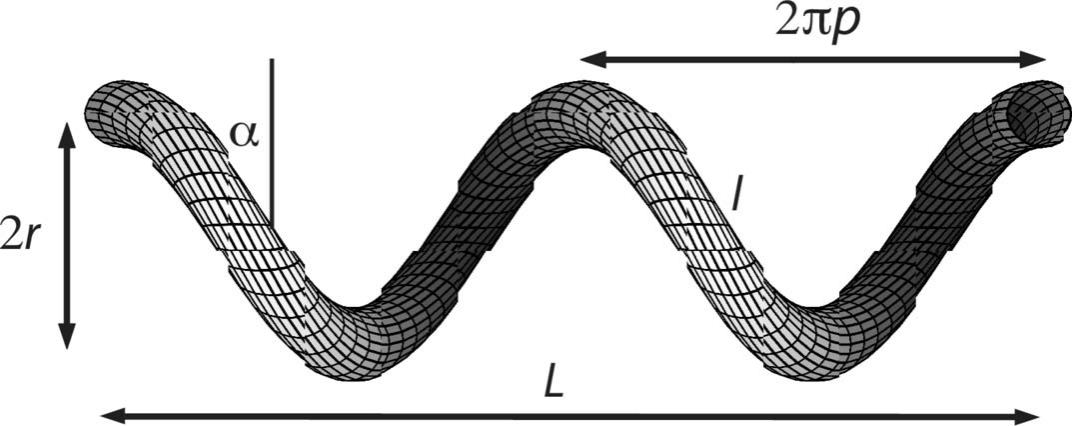
A single helix with radius *r*, pitch 2π*p*, strand contour length *l* and *z*-axis projected helix length *L*. In this specific example, the helix length *L* equals two times the helical repeat, but in general *L* is a variable parameter depending on *r*, *p* and *l* through equation (1).

Consider a single helix with radius *r*, pitch 2π*p* and length along its contour *l*, as illustrated in Fig. 1 ▸. The length of the helix projected on its helical axis is denoted by *L*. The structure of the helix is fully characterized by *p*, *r* and *l* (the effect of the finite cross section of the strand will be considered below). From integration along the contour it follows that 

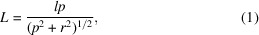

and the pitch angle α is defined through 

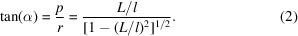

For the calculation of the form factor, the helix is placed with its central axis along the *z* axis in a coordinate system with Cartesian unit vectors **i**, **j** and **k**. A point on a right-handed helix is then described by position vector 

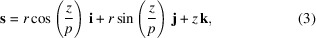

with *z* the projected distance on the helical axis. The momentum-transfer vector **q** is expressed in terms of its magnitude *q* = |**q**| and spherical coordinates θ and φ, so that 

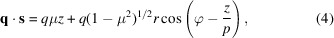

with 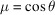 . The scattering amplitude 

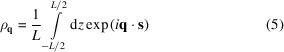

(with complex conjugate 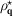 ) then takes the form 

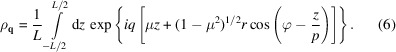

With the help of the associated series of Bessel functions of integer order *J*_*k*_, 

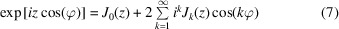

(Abramowitz & Stegun, 1970 ▸; Gradshteyn & Ryzhik, 1980 ▸), the scattering amplitude can be expressed as 

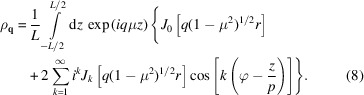

After integration of *z* over the length of the helical axis *L*, one obtains 

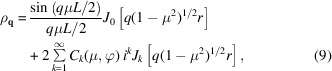

with the orientation-dependent coefficients 

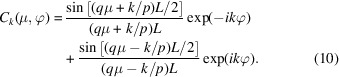

For a left-handed helix, the corresponding coefficients are given by the complex conjugate 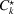 . The average of *C*_*k*_ over the azimuthal angle φ is identically zero, that is 

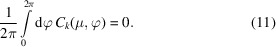

Furthermore, the coefficients *C*_*k*_ are orthogonal, 

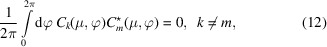

and normalized according to 

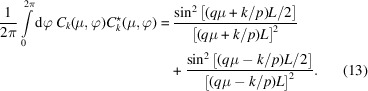




The form factor is given by 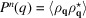 , where the angle brackets denote an isotropic orientation average of the momentum-transfer vector with respect to the helical axis (Lovesey, 1984 ▸). The index *n* denotes the number of strands per helix (for a single helix, *n* = 1). An isotropic average involves integration over azimuthal angle φ and polar coordinate μ according to 

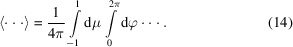

The form factor can now be calculated by isotropic averaging of the product of the scattering amplitude [equation (9)] with its complex conjugate and using the properties of the orientation-dependent coefficients [equations (11), (12) and (13)]. The exact expression of the form factor then takes the form 

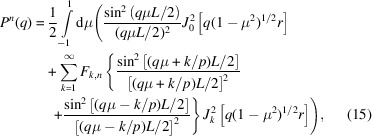

where the final integration of the orientation parameter μ has to be done numerically. In the presence of partial orientation order, the latter integration can be carried out by including an orientation distribution function. A factor *F*_*k*,*n*_ is introduced to facilitate the formulation of the form factors pertaining to higher-order helices with *n* > 1 (see below). In the case of a single helix, *n* = 1 and *F*_*k*,1_ = 1 for all values of index *k*. The form factor is normalized to unity for *q* = 0.

In the long-wavelength limit *q* → 0, the form factor can be expanded in powers of *q* according to 

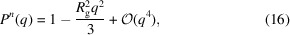

with radius of gyration *R*_g_. In the expansion of equation (15), the term proportional to 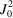 gives 

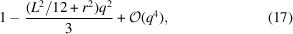

whereas the term proportional to 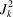 with index *k* = 1 contributes 

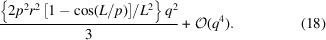

Terms proportional to 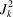 with index *k* > 1 contribute to the fourth and higher powers of *q* and are, hence, irrelevant in the derivation of second moment *R*_g_. Accordingly, one obtains 

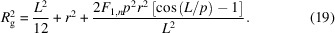

The third term on the right-hand side vanishes if the length of the helix *L* matches an exact multiple of the pitch 2π*p*. The third term also vanishes for diametrically symmetric helices with number of strands *n* > 1 (see below).

In practice, often local helical structure is probed with *qL* ≫ 1. Note that π/(*qL*) is the expression of the form factor of a rod with length *L* in the *qL* ≫ 1 limit. Accordingly, in order to derive the corresponding limiting expression for the helix, we will evaluate the normalized form factor 

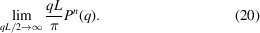

With the help of the following representation of the Dirac delta function, 

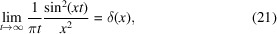

and the exact expression of the form factor [equation (15)], we obtain 

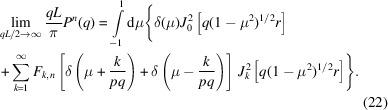

The integration over μ gives μ = 0 and μ = *k*/*pq* for the terms proportional to 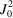 and 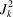 , respectively. Furthermore, the summation index *k* ≤ *pq* because the orientation variable is 0 ≤ μ ≤ 1. Accordingly, for values of *q* far exceeding the inverse length of the helix, one obtains the high-*q* limiting form 

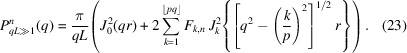




**Figure 2:**
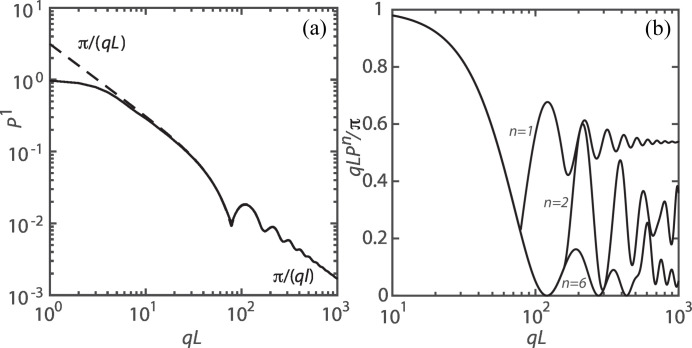
(*a*) The form factor of a single helix according to the exact (solid curve) and limiting (dashed curve) formulas. (*b*) The normalized form factor *qLP* ^*n*^/π pertaining to single (*n* = 1), diametrically symmetric (*n* = 2) and sixth-order (*n* = 6) helices. Notice that *qLP* ^*n*^/π tends to *L*/(*nl*) for *qL* → ∞. For both (*a*) and (*b*), the parameters are radius *r* = 5, pitch 2π*p* = 20 and helix length *L* = 250.

The exact and limiting form-factor formulas pertaining to a single helix, equations (15) and (23) with *n* = 1, respectively, are compared in Fig. 2 ▸(*a*). They are indiscernible for, say, *qL* > 10. At very high values of *qL*, the scattering is given by a rod-like segment of the strand with form factor π/(*ql*) (*l* being the contour length of the strand). In the intermediate *qL* range, the structure factor shows oscillatory behaviour. This oscillatory behaviour is more clearly shown by the normalized form factor *qLP* ^1^/π in Fig. 2 ▸(*b*). Variation of the radius and pitch gives a shift in the positions of the maxima and minima but no qualitative change in oscillatory behaviour.

In the derivation of the form factor, the effect of the finite cross section of the strand has been ignored. Because of the difference in length scales, this effect can be taken into account by multiplication of the form-factor formulas with that pertaining to the cross section *P*_c_(*q*). In the case of a Gaussian radial strand profile with second moment 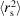 , the cross-sectional form factor is given by 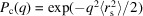 .

## 3. Double helix

**Figure 3:**
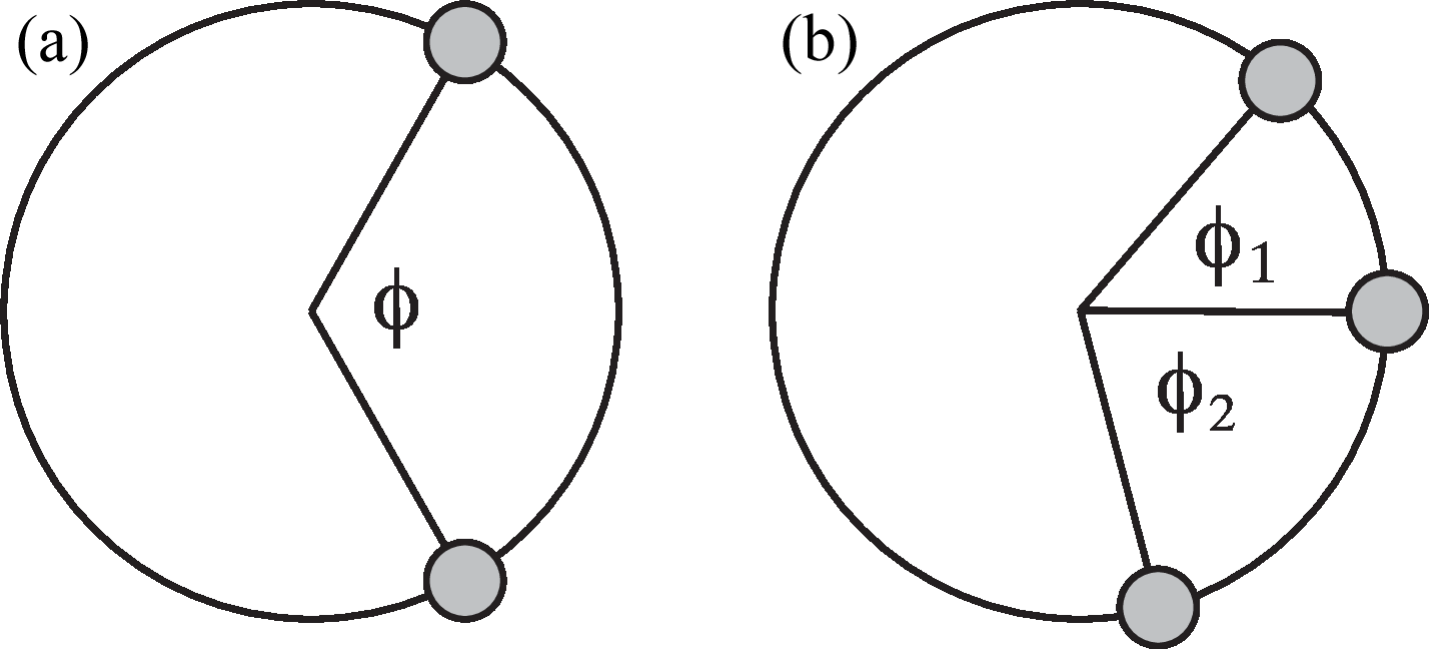
(*a*) The transverse cross section of a double helix with enclosed azimuthal angle ϕ. (*b*) As in (*a*) but for a triple helix with azimuthal angles ϕ_1_ and ϕ_2_.

In the case of a double helix, one needs to average the scattering amplitudes pertaining to the two individual coaxial strands. Let the azimuthal angle between the two strands be ϕ [see Fig. 3 ▸(*a*)]. A point on strand ± is then described by position vector 

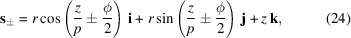

so that 

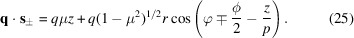

We first consider the situation of two diametrically opposed strands with ϕ = π. The calculation of the scattering amplitude involves averaging of the orientation-dependent coefficients pertaining to the two strands, that is 

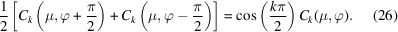

The derivation of the form factor closely follows that for the single helix with almost identical results, but now with factor 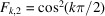 (*n* = 2). In this specific case, the summation is restricted to the terms with index *k* being even. As we will see below, the restriction of index *k* terms to multiples of the number of strands *n* is a common feature for diametrically symmetric helices with constant azimuthal-angle spacing between successive strands. A comparison with the result for the single helix is shown in Fig. 2 ▸(*b*). The double-helical structure exhibits much stronger oscillatory behaviour, coming from interference of the two opposing strands.

For any value of the enclosed azimuthal angle ϕ, the average of the orientation-dependent coefficients takes the form 

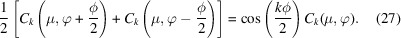

Again, the resulting formulas for the form factor *P* ^2^(*q*) and its high-*q* limiting form 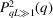 are almost identical to those for the single helix, but now with factor 

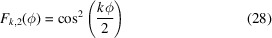

depending on the enclosed angle ϕ. This general result applies to helices exhibiting a major and minor groove, such as DNA in double-stranded form. With the width of the major and minor groove being *w* ^+^ and *w* ^−^, respectively, one obtains 

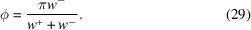

**Figure 4:**
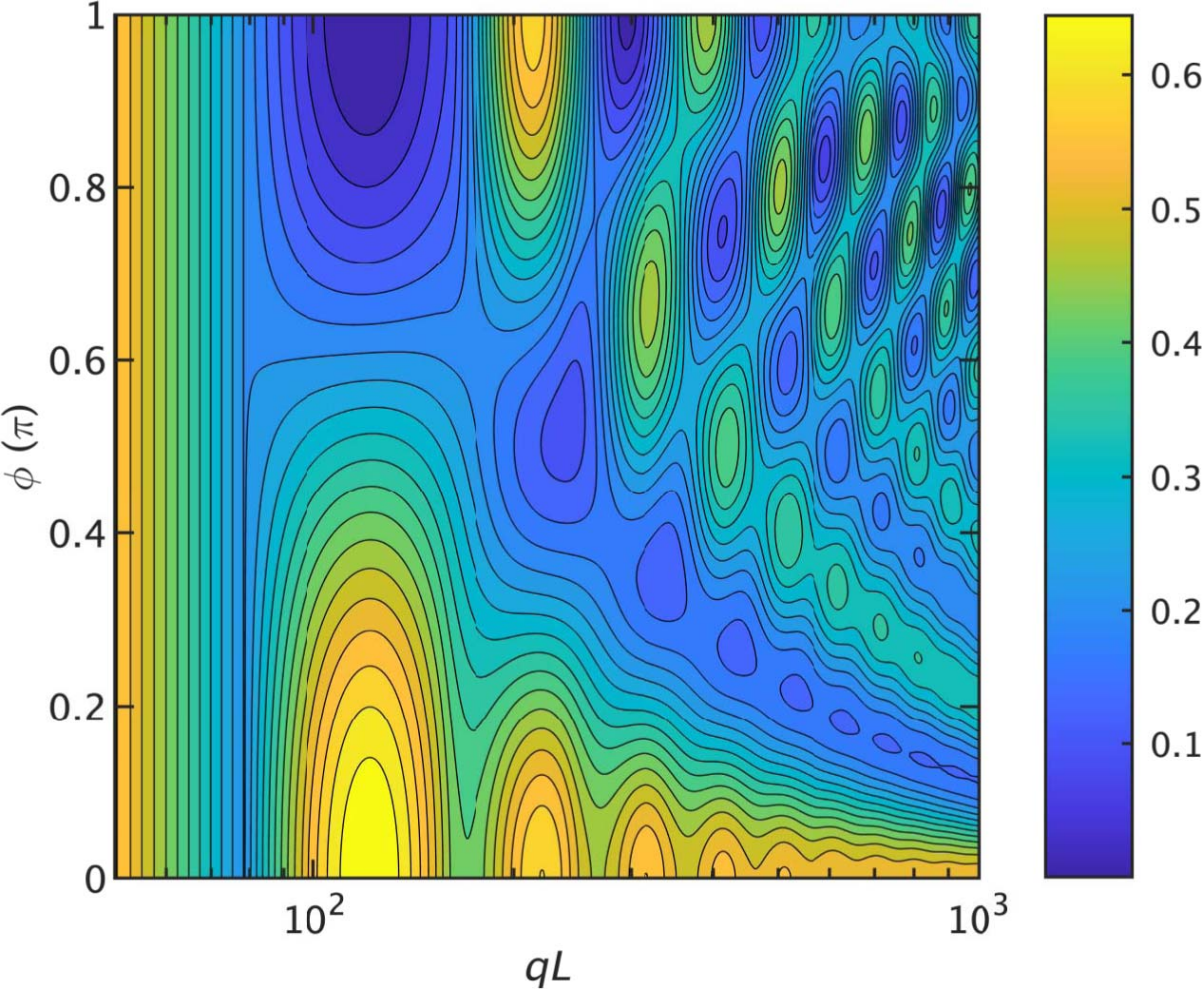
A contour plot of the normalized form factor *qLP* ^2^/π pertaining to a double helix with major and minor grooves with variable enclosed angle ϕ. The parameters are radius *r* = 5, pitch 2π*p* = 20 and helix length *L* = 250.

The effect of the presence of grooves of different width, through variation of ϕ, is illustrated by a contour plot of the normalized form factor *qLP* ^2^/π in Fig. 4 ▸. For ϕ = 0 and π, the results for the single and diametrically symmetric double helix are recovered [also shown in Fig. 2 ▸(*b*)]. For intermediate values of ϕ, the characteristic oscillation changes with gradual shifts in the positions of the maxima and minima.

## 4. Triple helix

A triple helix, composed of three strands, is characterized by two azimuthal angles (ϕ_1_ and ϕ_2_) [see Fig. 3 ▸(*b*)]. We will first consider the easier-to-handle situation with ϕ_1_ = ϕ_2_ = ϕ. The averaging of the orientation-dependent coefficients over the three strands in the calculation of the scattering amplitude then takes the form 

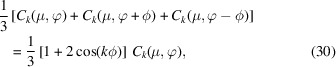

and subsequent calculation of the form factor gives 

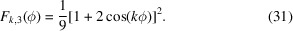

**Figure 5:**
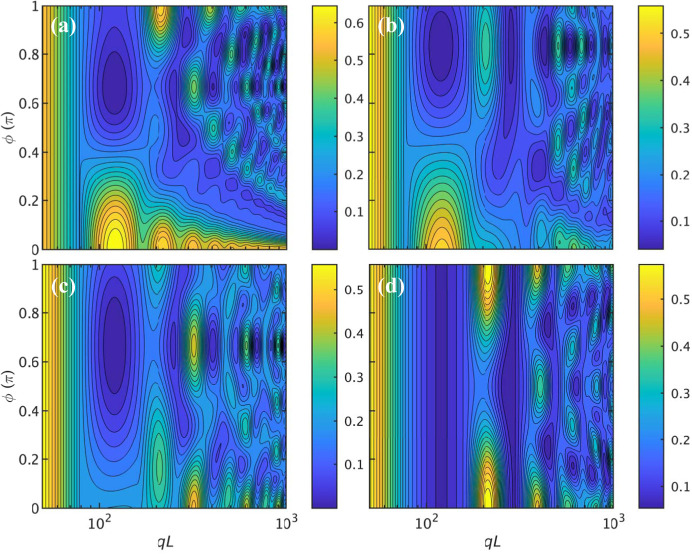
(*a*) A contour plot of the normalized form factor *qLP* ^3^/π pertaining to a triple helix with variable enclosed angle ϕ_1_ = ϕ_2_ = ϕ. (*b*)–(*d*) As in (*a*) but for the general case with variable angle ϕ_1_ and fixed angles ϕ_2_ = π/3, 2π/3 and π, respectively. For all plots, the parameters are radius *r* = 5, pitch 2π*p* = 20 and helix length *L* = 250.

For a diametrically symmetric triple helix with ϕ = 2π/3, the summation is restricted to values of *k* being multiples of 3. A contour plot of the normalized form factor *qLP* ^3^/π is shown in Fig. 5 ▸(*a*). The positions of the minima and maxima are similar to those of the double helix, shown in Fig. 4 ▸. Furthermore, the form factors for the single and diametrically symmetric double helix are recovered for ϕ = 0 and π, respectively.

In the general case with different angles ϕ_1_ and ϕ_2_, the average of the orientation-dependent coefficients cannot be expressed in terms of a single coefficient *C*_*k*_(μ, φ). It can however be written as the sum of two coefficients with different azimuthal angles as 

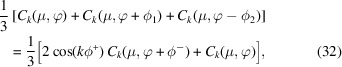

with ϕ^+^ = (ϕ_1_ + ϕ_2_)/2 and ϕ^−^ = (ϕ_1_ − ϕ_2_)/2. The form factor can still be calculated in the usual way, but now one needs the additional orthogonality and normalization properties 

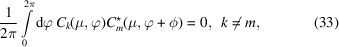

and 

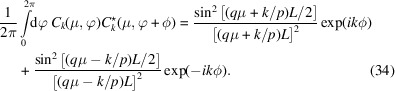

The resulting expressions for *P* ^3^(*q*) and 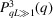 are almost identical to those for the single helix but with factor 

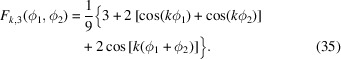

As in the case of the double helix, this general expression takes into account the possible presence of grooves of different widths. Furthermore, it reduces to equation (31) for the situation with ϕ_1_ = ϕ_2_ = ϕ.

The effect of the asymmetry of the triple helix, through variation of ϕ_1_ but fixed ϕ_2_, is illustrated by contour plots of the normalized form factor *qLP* ^3^/π in Figs. 5 ▸(*b*)–5 ▸(*d*). Rather complex behaviour is observed with a strong dependence of the positions of the maxima and minima on the cross-sectional distribution of the strands.

## 5. Higher-order helices

We now consider a set of *n* coaxial strands. The set is twisted about its central axis and diametrically symmetric with a constant spacing of 2π/*n* in azimuthal angle between successive strands. Owing to its symmetry, the average of the orientation coefficients in the scattering amplitude can be expressed in terms of a single coefficient *C*_*k*_(μ, φ) according to 

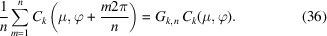

With equation (10), one readily obtains

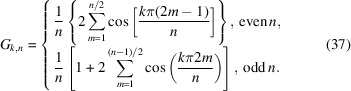

For number of strands *n* in the range 2–6, the results are collected in Table 1 ▸. Results for the diametrically symmetric double and triple helices with *n* = 2 and 3, respectively, agree with those derived in previous sections. Evaluation of equation (37) as well as the entries in Table 1 ▸ shows that 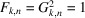 for index *k* being multiples of *n*, and *F*_*k*,*n*_ = 0 otherwise. This implies that, for a diametrically symmetric helix composed of *n* strands with a constant azimuthal spacing of 2π/*n*, the index *k* summation in the form-factor expression is restricted to the *nk* terms, that is even *k* for *n* = 2, *k* being a multiple of 3 for *n* = 3 *etc*.

The normalized form factors for the single, diametrically symmetric double and sixth-order helices are compared in Fig. 2 ▸(*b*). With increasing number of strands *n*, the normalized form factor exhibits increasingly irregular oscillatory behaviour and tends to lower values *L*/(*nl*) for *qL* → ∞.

Following expansion of the form factor in powers of *q*, one obtains the radius of gyration of the diametrically symmetric helix as

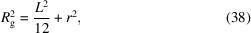

which depends on the radius and length but not on the number of strands (recall that *k* ≥ *n* and 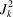 terms with *k* > 1 do not contribute to the second moment).

## 6. Twisted fibres

**Figure 6:**
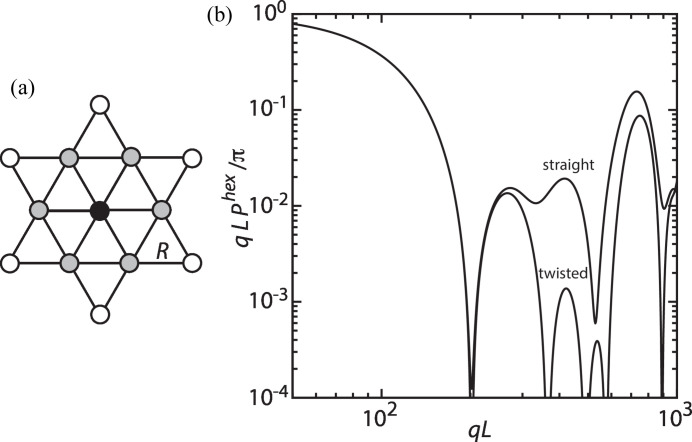
(*a*) The transverse cross section of a hexagonal fibre. Note that there is one strand at the core (black), six strands at a radial distance *R* (grey) and another six strands at distance 3^1/2^ *R* (white). (*b*) The normalized form factor *qLP* ^hex^/π of a twisted (pitch 2π*p* = 20) and straight (2π*p* = ∞) fibre with a cross section as illustrated in (*a*). The other parameters are *R* = 5 and fibre length *L* = 500.

So far, we have considered helical structures with an equal radial distance *r* from the central axis for all strands. However, fibres are generally made of a twisted bundle of strands with a range of radial distances. As an example, we will derive the form factor of a hexagonal fibre with a transverse cross section as illustrated in Fig. 6 ▸(*a*). In this particular example, there is one strand at the core, six strands at a radial distance *R* and another six strands at distance 3^1/2^ *R* (a total of 13 strands). All off-centre strands have the same helical pitch but their curvature depends on *R*. The strand at the core is in a straight configuration.

The scattering amplitude of the fibre can be calculated by taking the weighted average of the scattering amplitudes pertaining to the strands at the core and the two radial distances, that is 

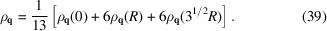

For the strand at the core, the scattering amplitude is simply given by 

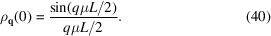

The sets of six strands (*n* = 6) at radial distances *R* and 3^1/2^ *R* are diametrically symmetric with an azimuthal-angle spacing of π/3. Furthermore, the set of strands at distance 3^1/2^ *R* is rotated by a phase factor of π/6 with respect to the set at distance *R*. With the help of the results obtained in the previous section, the corresponding scattering amplitudes take the forms 

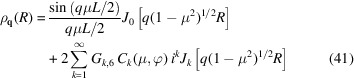

and 

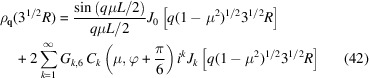

The *G*_*k*,6_ term restricts the index *k* summation to multiples of 6. The form factor of the hexagonal fibre can now be calculated in the usual way. The result is given by 

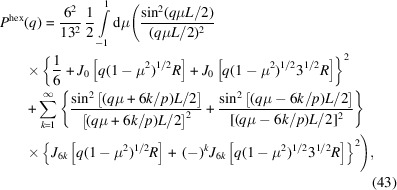

with its high-*q* limiting form 

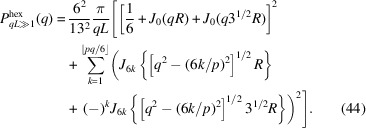

Normalized form factors *qLP* ^hex^/π for a twisted and straight hexagonal fibre are shown in Fig. 6 ▸(*b*). The effect of twisting comes to the fore at higher *qL* values. In the *qL* range up to the first maximum, it is sufficient to include the *J*_0_ terms only.

The radius of gyration of the hexagonal fibre does not depend on the pitch and is given by 

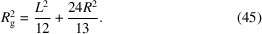

For fibres of different symmetry and/or different number of concentric layers of strands, the formalism can easily be adapted by taking the weighed average of the relevant layer’s scattering-length amplitudes (depending on radial distance, number of strands and relative orientation).

## 7. Conclusions

Formulas have been presented for the isotropically averaged single-chain scattering function (form factor) of single, double, triple and higher-order helices, and a twisted hexagonal fibre. Form factors of double and triple helices with grooves of different widths have also been derived. The formulas include the longitudinal and transverse interference over the pitch and radius of the helices, respectively. Limiting equations valid for values of momentum transfer far exceeding the inverse length of the helical structures have also been presented. The results are cast in a general formalism for easy adaptation of the number of strands per helix and cross-sectional symmetry. Furthermore, the formalism pertaining to the fibre can easily be adapted for fibres of different symmetry and/or number of concentric layers of strands. The form-factor expressions may be useful for the analysis of small-angle scattering data of randomly or partially oriented (bio)macromolecules or molecular assemblies exhibiting helical structural arrangements.

## Figures and Tables

**Table 1:** Factor *G*_*k*,*n*_ for symmetric helices composed of *n* strands with azimuthal-angle increment 2π/*n* for *n* in the range 2–6

*n*	*G*_*k*,*n*_
2	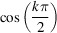
3	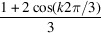
4	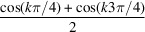
5	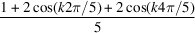
6